# AMBRA1 Negatively Regulates the Function of ALDH1B1, a Cancer Stem Cell Marker, by Controlling Its Ubiquitination

**DOI:** 10.3390/ijms222112079

**Published:** 2021-11-08

**Authors:** Seung-Heon Baek, Yeun-Kyu Jang

**Affiliations:** 1 Department of Systems Biology, College of Life Science and Biotechnology, Yonsei University, Seoul 03722, Korea; baeksh9505@naver.com; 2 BK21 Yonsei Education & Research Center for Biosystems, Yonsei University, Seoul 03722, Korea

**Keywords:** colorectal cancer stem cell, E3 ubiquitin ligase, substrate receptor, aldehyde dehydrogenase 1B1, noncanonical ubiquitination

## Abstract

Activating molecule in Beclin-1-regulated autophagy (AMBRA1), a negative regulator of tumorigenesis, is a substrate receptor of the ubiquitin conjugation system. ALDH1B1, an aldehyde dehydrogenase, is a cancer stem cell (CSC) marker that is required for carcinogenesis via upregulation of the β-catenin pathway. Although accumulating evidence suggests a role for ubiquitination in the regulation of CSC markers, the ubiquitination-mediated regulation of ALDH1B1 has not been unraveled. While proteome analysis has suggested that AMBRA1 and ALDH1B1 can interact, their interaction has not been validated. Here, we show that AMBRA1 is a negative regulator of ALDH1B1. The expression of ALDH1B1-regulated genes, including *PTEN*, *CTNNB1* (β-catenin), and CSC-related β-catenin target genes, is inversely regulated by AMBRA1, suggesting a negative regulatory role of AMBRA1 in the expression of ALDH1B1-regulated genes. We found that the K27- and K33-linked ubiquitination of ALDH1B1 is mediated via the cooperation of AMBRA1 with other E3 ligases, such as TRAF6. Importantly, ubiquitination site mapping revealed that K506, K511, and K515 are important for the K27-linked ubiquitination of ALDH1B1, while K33-linked ubiquitination occurs at K506. A ubiquitination-defective mutant of ALDH1B1 increased the self-association ability of ALDH1B1, suggesting a negative correlation between the ubiquitination and self-association of ALDH1B1. Together, our findings indicate that ALDH1B1 is negatively regulated by AMBRA1-mediated noncanonical ubiquitination.

## 1. Introduction

Activating molecule in Beclin-1-regulated autophagy (AMBRA1) is a highly intrinsically disordered protein. As with other intrinsically disordered proteins [1], AMBRA1 is involved in various biological processes and regulates protein–protein interactions [2]. AMBRA1 is involved in the induction of autophagy via interactions with ULK1 [3] and elongin B [4] and in the induction of mitophagy via interactions with PARKIN [5] and LC3 [6]. Furthermore, AMBRA1 is crucial for nervous system development in mouse embryos [7] and skeletal muscle development in zebrafish [8].

Recently, AMBRA1 has emerged as a tumor suppressor that mediates the degradation of proto-oncogene c-Myc [9] and D-type cyclins [10]. AMBRA1-deficient tumor cells were more likely to grow when injected into nude mice than wild-type tumor cells, implying a negative relationship between AMBRA1 and tumorigenesis [11]. In addition, AMBRA1 expression inversely correlates with the stemness signature in lung cancer [12]. Despite the increasing evidence of the functional role of AMBRA1 in cancer stem cell (CSC) properties, the underlying mechanisms remain unknown.

Aldehyde dehydrogenases (ALDHs) are members of a superfamily of NAD(P)^+^-dependent enzymes and catalyze the oxidation of aldehydes to their respective acids. ALDH1B1, which is one of the ALDH isozymes, is reported to be strictly expressed in the SC compartments of normal human colon and is highly expressed in human colonic adenocarcinoma [13]. In a clinical study [14], the upregulation of ALDH1B1 was correlated with high-grade colorectal carcinoma and the presence of lymph-node metastases, implying a potential role of ALDH1B1 in carcinogenesis. The depletion of ALDH1B1 in colon cancer cells resulted in the downregulation of Wnt/β-catenin, Notch, and PI3K/Akt pathway-related genes such as *CTNNB1*, *Akt*, and *Notch1* [15]. Negative regulation of ALDH1B1 by microRNA-761 in osteosarcoma cells suppresses tumor cell proliferation by regulating TGF-β signaling and cell adhesion [16]. Although several lines of evidence suggest the importance of the modification and regulation of CSC markers by ubiquitination [17], few studies have investigated the regulators modifying ALDH1B1.

According to the Biological General Repository for Interaction Datasets (thebiogrid.org, Accessed 15 December 2020), ALDH1B1 has been identified as one of the AMBRA1-interacting proteins via affinity purification-mass spectrometry analysis of Flag-AMBRA1-transfected HEK293T human embryonic kidney cells [18]. However, the biological significance of the interaction between AMBRA1 and ALDH1B1 is not yet understood. Here, for the first time, we show that AMBRA1 downregulates the self-association and enzyme activity of ALDH1B1, and subsequently regulates CSC-associated Wnt/β-catenin signaling in an ALDH1B1-dependent manner. Mechanistically, AMBRA1 controls ALDH1B1 function via the ubiquitination of ALDH1B1 with K27- and K33-linked ubiquitin chains. Further, we found that TRAF6 and DDB1, an adaptor protein of the CRL4 complex, are able to ubiquitinate ALDH1B1 in an AMBRA1-independent fashion.

## 2. Results

### 2.1. AMBRA1 Interacts with ALDH1B1 and Suppresses the Self-Association of ALDH1B1

**Figure 1:**
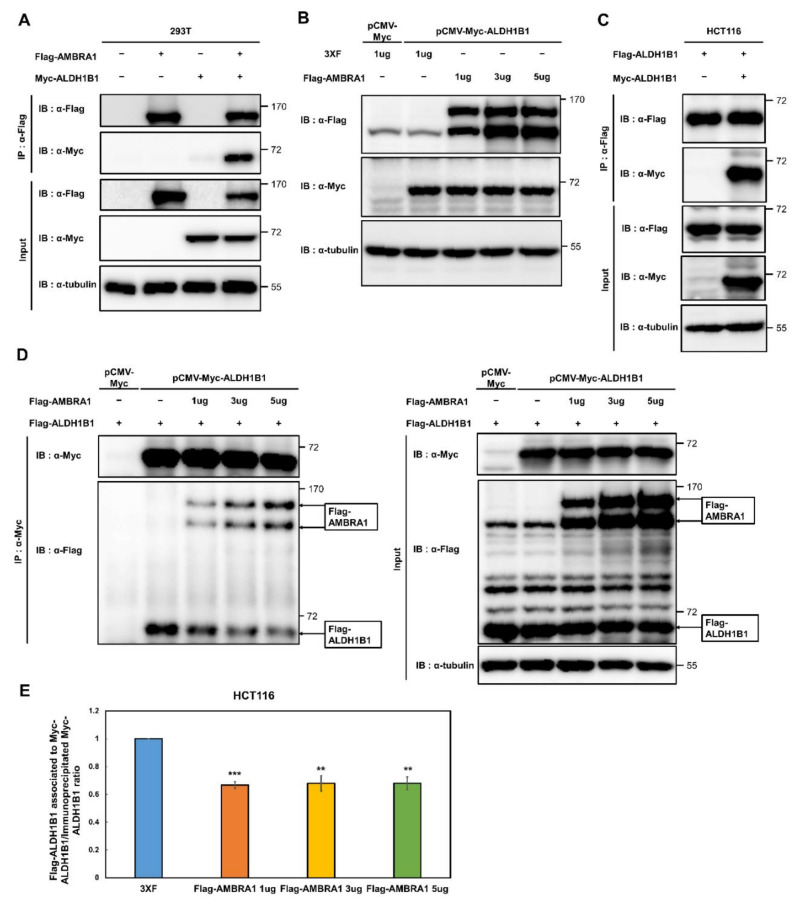
AMBRA1 regulates the self-association, but not the expression, of ALDH1B1. (**A**) The interaction between Flag-tagged AMBRA1 (Flag-AMBRA1) and Myc-tagged ALDH1B1 (Myc-ALDH1B1) was identified by a co-immunoprecipitation (co-IP) assay in HEK293T cells. α-Tubulin was detected as a loading control (*n* = 5). (**B**) Protein expression of Myc-ALDH1B1 after overexpression of indicated amounts of 3×F vector (pCMV-3Tag-6, empty vector) and Flag-AMBRA1, as measured by immunoblot analysis. The 3×F vector and Flag-AMBRA1 were transfected into HCT116 cells that stably overexpressed Myc-ALDH1B1 (pCMV-Myc-ALDH1B1 HCT116 cells) (*n* = 3). (**C**) The interaction between Flag-tagged ALDH1B1 (Flag-ALDH1B1) and Myc-tagged ALDH1B1 (Myc-ALDH1B1) was confirmed by immunoprecipitation and immunoblot analysis (*n* = 3). (**D**) The empty vector and Flag-AMBRA1 were cotransfected with Flag-ALDH1B1 into pCMV-Myc-ALDH1B1 HCT116 cells. Then, co-IP was performed using anti-c-Myc agarose affinity gel antibody and western blotting was conducted using anti-Flag, anti-Myc, and anti-α-tubulin antibodies (*n* = 3). (**E**) Quantification of the results in (**D**) using the ImageJ software. Protein levels of Flag-ALDH1B1 in immunoprecipitate samples were normalized to that of Myc-ALDH1B1. The ratio of the control (3×F) was set at 1. ** *p* < 0.01, *** *p* < 0.001 vs. control.

A recent study showed that the interactome of AMBRA1 contains ALDH1B1 [18]; however, ALDH1B1 may not be of interest as an AMBRA1 interactor given its low interaction score and the fact that their interaction has not yet been confirmed. To evaluate whether AMBRA1 indeed interacts with ALDH1B1, we performed a co-immunoprecipitation (Co-IP) assay using Flag-tagged AMBRA1 and Myc-tagged ALDH1B1 in HEK293T cells, which confirmed their physical interaction (Figure 1A). Next, we examined the effect of AMBRA1 on the protein stability of ALDH1B1 through this interaction. AMBRA1 is known to regulate the protein stability of its interaction partners. ULK1 is stabilized by AMBRA1-mediated ubiquitination [3], whereas AMBRA1 enhances the proteasomal degradation of target proteins such as cyclin D and elongin C [10,11,12,18]. To analyze the dose-dependent effect of AMBRA1 expression on steady-state ALDH1B1 protein levels, AMBRA1 was ectopically expressed in a dose-dependent manner in HCT116 human colorectal cancer cells that stably overexpressed Myc-ALDH1B1. The data showed that ALDH1B1 levels were little affected by AMBRA1 overexpression (Figure 1B).

Thus, we next questioned whether AMBRA1 affects the interaction between ALDH1B1 and its interaction partners, including itself. According to a structural modeling study of ALDH1B1, ALDH1B1 is capable of interacting with another ALDH1B1 [19]. However, as the self-association of ALDH1B1 has been only demonstrated by computational modeling, we decided to investigate the self-association by a Co-IP assay using differentially tagged ALDH1B1 (Figure 1C). To examine the effect of AMBRA1 overexpression on the self-association of ALDH1B1, increasing doses of Flag-tagged AMBRA1 were cotransfected with Flag-tagged ALDH1B1 into HCT116 cells stably expressing Myc-tagged ALDH1B1. The results revealed that the protein levels of Flag-tagged ALDH1B1 in immunoprecipitates were decreased by AMBRA1 overexpression, suggesting that overexpressed AMBRA1 interfered with the interaction between Flag-ALDH1B1 and Myc-ALDH1B1 (Figure 1D). We quantified anti-Flag-ALDH1B1 and anti-Myc-ALDH1B1 based on immunoblots of the immunoprecipitates, using ImageJ. The data showed that the rate of Flag-ALDH1B1 association with Myc-ALDH1B1 in immunoprecipitated Myc-ALDH1B1 samples was significantly reduced by Flag-AMBRA1 overexpression, in a dose-independent manner (Figure 1E). Together, these results demonstrated that AMBRA1 can associate with ALDH1B1 and that this interaction interferes with the self-association of ALDH1B1, suggesting that AMBRA1 acts as a negative regulator of ALDH1B1.

### 2.2. The PTEN mRNA Level Is Reversely Regulated by ALDH1B1 and AMBRA1

**Figure 2:**
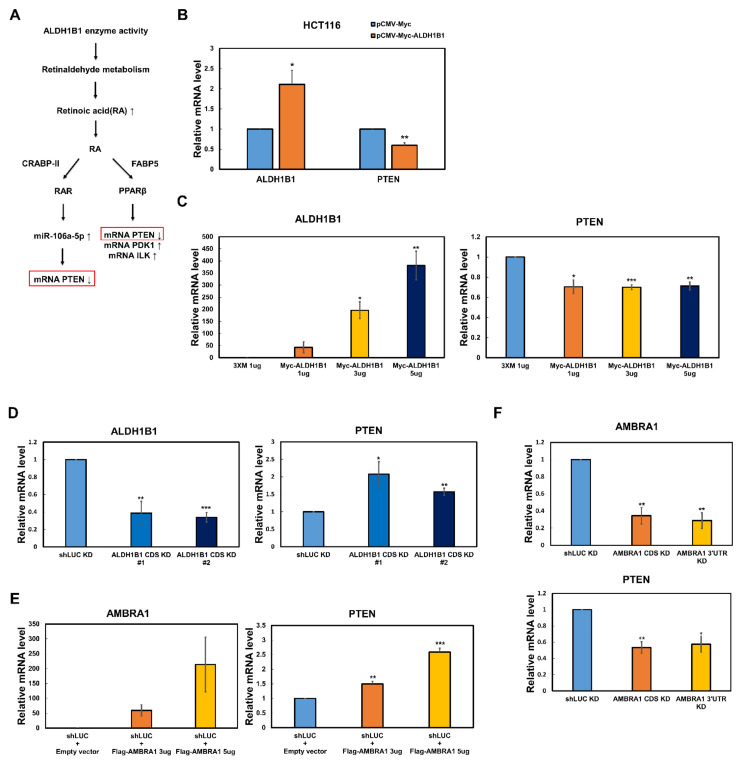
ALDH1B1 and AMBRA1 inversely regulate *PTEN* transcription. (**A**) Schematic flow diagram of the enzyme activity of ALDH1B1 regulating the transcription of PTEN. (**B**) mRNA levels of *ALDH1B1* and *PTEN* in HCT116 cell lines stably expressing Myc-ALDH1B1, as assessed by RT-qPCR (*n* = 4). (**C**) mRNA levels of *ALDH1B1* and *PTEN* in HCT116 cells overexpressing the indicated amounts of 3×M vector (pCMV-3Tag-2, empty vector) and Myc-ALDH1B1, as assessed by RT-qPCR (*n* = 3). (**D**) mRNA levels of *ALDH1B1* and *PTEN* in control (shLUC) and *ALDH1B1*-depleted (shALDH1B1 #1, #2) HCT116 cells, as assessed by RT-qPCR (*n* = 3). Symbols: shLUC KD, control knockdown; ALDH1B1 CDS KD, knockdown targeting the coding region of *ALDH1B1*; 3’ UTR KD, knockdown targeting 3’ UTR region. (**E**) mRNA levels of *AMBRA1* and *PTEN* in HCT116 cells transiently overexpressing empty vector or the indicated amounts of Flag-AMBRA1, as assessed by RT-qPCR (*n* = 3). (**F**) mRNA levels of *AMBRA1* and *PTEN* in *AMBRA1*-deficient HCT116 cells, as assessed by RT-qPCR (*n* = 3). In (**B**–**F**), mRNA levels were normalized to that of *GAPDH* and the expression levels in the control were set at 1. * *p* < 0.05, ** *p* < 0.01, *** *p* < 0.001 vs. control.

ALDH1B1 has high sequence similarity with all homotetrameric mammalian ALDHs [19]. As expected, a 3D model of human ALDH1B1 revealed that the functional unit of ALDH1B1 is a homotetramer. In the above Co-IP assay, AMBRA1 negatively affected the self-association of ALDH1B1, suggesting that AMBRA1 can disrupt the functional unit of ALDH1B1 and, thus, its enzyme activity. We found in the literature that the mRNA level of *PTEN* reflects ALDH1B1 enzyme activity [15,20,21]. Homotetrameric ALDH1B1 metabolizes retinaldehyde, thereby generating retinoic acid (RA). RA can bind two lipid-binding proteins, cellular RA-binding protein-II (CRABP-II) and fatty acid-binding protein5 (FABP5), which transport RA from the cytosol to the nucleus and activate two ligand-activated transcription factors, RA receptor (RAR) and PPARβ/δ, respectively [15]. RA-mediated RAR activation leads to the transcriptional repression of *PTEN* through miR-106a-5p in mouse embryonic palatal mesenchymal cells [20] and PPARβ directly downregulates *PTEN* transcription in keratinocytes [21] (Figure 2A). As the oxidative enzyme activity of ALDH1B1 suppresses the mRNA level of *PTEN* via either way, we questioned whether ALDH1B1 regulates *PTEN* transcription in HCT116 cells. Among the available colorectal cancer cell lines, HCT116 cells were selected for studying ALDH1B1 enzyme activity because these cells constitutively and highly express ALDH1B1, while they show low ALDH1A1 expression [22]. As ALDH1A1 is reportedly associated with the stemness of several tumors via RA generation [23], the use of HCT116 as a host cell line would minimize any confounding effect by ALDH1A1 [15]. The results showed that the overexpression of ALDH1B1 led to decreased mRNA levels of *PTEN*, irrespective of whether ALDH1B1 was stably or transiently expressed (Figure 2B,C). In contrast, when *ALDH1B1* was depleted using small hairpin (sh)RNAs targeting two different coding sequences of *ALDH1B1*, *PTEN* transcription was enhanced (Figure 2D). These results were consistent with those in previous reports on ALDH1B1 enzyme activity [15,20,21]. To evaluate the effect of AMBRA1 expression on the mRNA level of *PTEN*, *PTEN* mRNA levels in HCT116 cells ectopically expressing AMBRA1 and *AMBRA1*-depleted HCT116 cells were measured using quantitative reverse transcription (RT-q) PCR. The data showed that, in contrast to ALDH1B1, AMBRA1 affected the *PTEN* mRNA level (Figure 2E,F). These findings supported that AMBRA1 disrupts the self-association of ALDH1B1, as shown in Figure 1.

### 2.3. AMBRA1 Regulates the Transcription of PTEN, CTNNB1, and CSC-Related β-Catenin Target Genes via ALDH1B1

**Figure 3:**
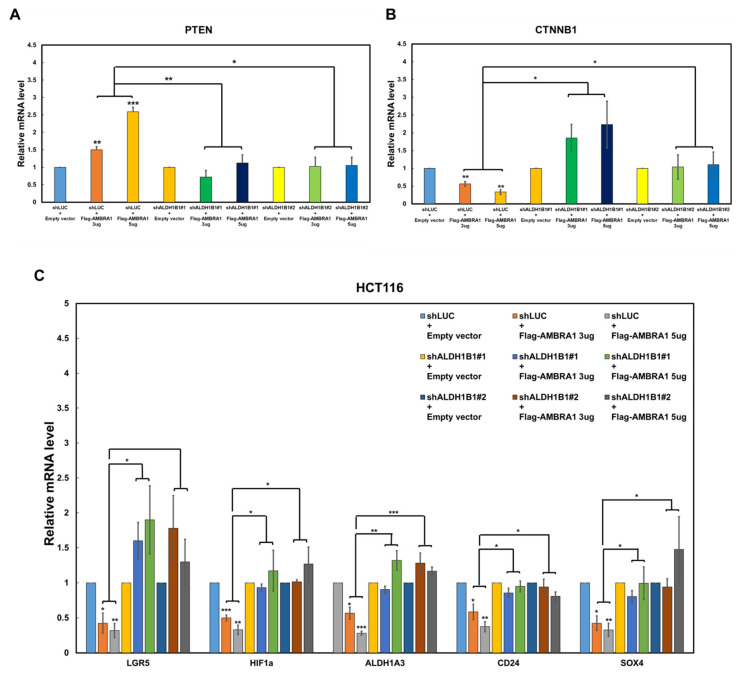
AMBRA1 affects the mRNA levels of *PTEN*, *CTNNB1* (β-catenin), and CSC-associated β-catenin target genes in an ALDH1B1-dependent manner. (**A**) mRNA levels of *PTEN* in wild-type (WT) and *ALDH1B1*-knockdown HCT116 cells overexpressing empty vector or the indicated amounts of Flag-AMBRA1, as assessed by RT-qPCR (*n* = 3). (**B**) mRNA expression levels of *CTNNB1* in control (shLUC) and *ALDH1B1*-depleted (shALDH1B1 #1, #2) HCT116 cells overexpressing empty vector or the indicated amounts of Flag-AMBRA1, as assessed by RT-qPCR (*n* = 3). (**C**) mRNA expression levels of β-catenin target genes associated with cancer stem cells (CSCs), as assessed by RT-qPCR using the same samples as those used in (**B**) (*n* = 3). In (**A**–**C**), mRNA expression levels were normalized to that of *GAPDH* and the mRNA levels in the control were set at 1. In WT HCT116 cells, mRNA levels after AMBRA1 overexpression were compared with the control. The average mRNA level after overexpressing AMBRA1 at the two indicated amounts in *ALDH1B1*-depleted HCT116 cells was compared with that in WT HCT116 cells. * *p* < 0.05, ** *p* < 0.01, *** *p* < 0.001 vs. control.

The above results implied that AMBRA1 upregulates *PTEN* transcription by affecting ALDH1B1. To investigate this, we overexpressed AMBRA1 in *ALDH1B1*-knockdown HCT116 cells and analyzed *PTEN* transcription. We observed that the stimulatory effect of AMBRA1 on *PTEN* transcription was lost in *ALDH1B1*-depleted cells (Figure 3A). A recent study showed that ALDH1B1 modulates the Wnt/β-catenin pathway, which is crucial for colorectal cancer formation [15]. According to the study, the knockdown of *ALDH1B1* in colorectal cancer cells resulted in a reduction in *CTNNB1* (β-catenin) mRNA levels. Based on this previous report, we determined whether the suppressive effect of AMBRA1 on ALDH1B1 self-association would lead to a reduction in *CTNNB1* mRNA levels. The overexpression of AMBRA1 reduced the *CTNNB1* mRNA level in parental HCT116 cells, but not in *ALDH1B1*-knockdown HCT116 cells (Figure 3B).

Accumulating evidence supports that the Wnt/β-catenin pathway plays important roles in CSCs as well as normal stem cells [24]. As AMBRA1 affected *CTNNB1* transcription via ALDH1B1, which is a crucial CSC marker of colorectal cancer, we next investigated whether AMBRA1 expression affects the mRNA levels of several CSC-related β-catenin target genes, including *LGR5*, *HIF1a*, *ALDH1A3*, *CD24*, and *SOX4*. Intriguingly, the results showed that these β-catenin target genes were significantly controlled by AMBRA1 and, more importantly, in an ALDH1B1-dependent manner (Figure 3C). Effective *AMBRA1* overexpression and *ALDH1B1* knockdown were confirmed by RT-qPCR (Supplementary Figure S1). Therefore, we concluded that AMBRA1 negatively modulates the stemness of colorectal cancer cells via the ALDH1B1-β–catenin axis.

### 2.4. ALDH1B1 Is Ubiquitinated by AMBRA1, DDB1, and TRAF6

**Figure 4:**
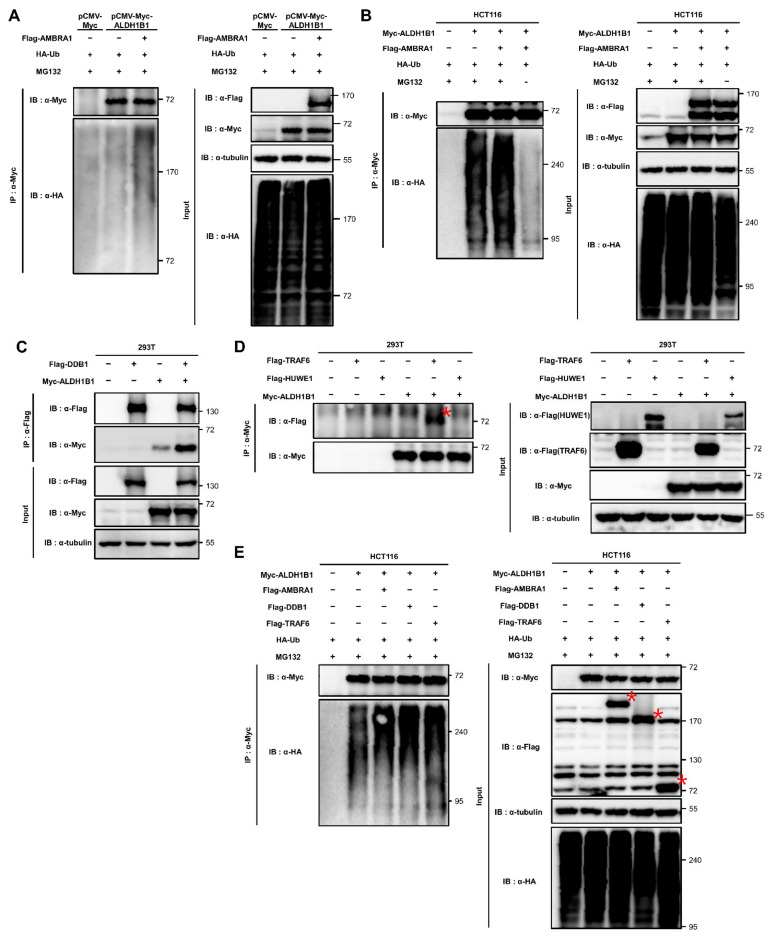
AMBRA1, DDB1, and TRAF6 are able to ubiquitinate ALDH1B1. (**A**) Empty vector (3×F vector) and Flag-tagged AMBRA1 (Flag-AMBRA1) were cotransfected with HA-tagged ubiquitin (HA-Ub) into pCMV-Myc-ALDH1B1 HCT116 cells. Ubiquitination of Myc-ALDH1B1 was analyzed by immunoprecipitating with anti-c-Myc agarose affinity gel antibody and detection by immunoblot analysis. Cells were treated with 10 μM MG132 (proteasome inhibitor) for 6 h. (**B**) Flag-AMBRA1 and Myc-ALDH1B1 were cotransfected with HA-Ub into HCT116 cells and the cells were harvested after 6 h of treatment with 10 μM MG-132 or control (DMSO) treatment. Immunoprecipitation with an anti-c-Myc agarose affinity gel antibody was used to measure the ubiquitination of Myc-ALDH1B1 (*n* = 3). (**C**) The interaction of Myc-ALDH1B1 with Flag-DDB1 was identified by Co-IP using anti-Flag affinity gel (*n* = 3). (**D**) After overexpressing Myc-ALDH1B1 with Flag-TRAF6 or Flag-HUWE1 in HEK293T cells, the interaction was investigated by immunoprecipitation and immunoblot analysis using an anti-c-Myc agarose affinity gel antibody. The asterisk indicates Flag-TRAF6 in the immunoprecipitates (*n* = 3). (**E**) The ubiquitination of Myc-ALDH1B1 after overexpression of Flag-AMBRA1, Flag-DDB1, and Flag-TRAF6 was analyzed by a cell-based ubiquitination assay and MG132 treatment (10 μM, 6 h) (*n* = 3). The red asterisks indicate the overexpressed proteins.

AMBRA1 is an E3 ligase adaptor that mediates ubiquitination by interacting with both the substrate and its E3 ligase [3,18,25]. As AMBRA1 interacts with ALDH1B1, we questioned whether AMBRA1 can enhance the ubiquitination of ALDH1B1. Flag-tagged AMBRA1 and HA-tagged ubiquitin (HA-Ub) were cotransfected into HCT116 cells stably expressing Myc-tagged ALDH1B1, followed by immunoprecipitation of Myc-ALDH1B1 and immunoblotting based on the HA tags. Before immunoprecipitation, the cells were treated with MG132, a proteasome inhibitor, as the poly-ubiquitination of targets leads to proteasomal degradation. The results showed that the levels of HA-Ub conjugated to Myc-ALDH1B1 increased after AMBRA1 overexpression, suggesting AMBRA1-mediated ubiquitination of ALDH1B1 (Figure 4A). Next, we questioned whether ubiquitinated ALDH1B1 is associated with the proteasome. As shown above, AMBRA1 did not affect the protein stability of ALDH1B1 and, therefore, we did not expect a relationship between ubiquitinated ALDH1B1 and the proteasome. However, when we excluded MG132 treatment in an in vivo ubiquitination assay, ubiquitinated ALDH1B1 levels were significantly diminished and even lower than those before as well as after AMBRA1 overexpression with MG132 treatment (Figure 4B). Thus, we predicted that ALDH1B1 can be ubiquitinated in an AMBRA1-independent manner through K11 or K48 linkages, which are hallmarks of proteasomal degradation [26]. Next, we conducted Co-IP experiments to identify AMBRA1-related E3 ligases that interact with ALDH1B1. AMBRA1, as an E3 ligase adaptor, directly interacts with several E3 ligases, including TRAF6 [3], CRL4-DDB1 complex [4], and HUWE1 [25]. Among these, we found that two E3 ligases, DDB1 and TRAF6, interact with ALDH1B1 (Figure 4C,D). To explore these interactions in detail, we conducted an in vivo ubiquitination assay. The results showed that ALDH1B1 ubiquitination was increased upon overexpression of DDB1 and TRAF6 as well as AMBRA1, suggesting the potential roles of DDB1 and TRAF6 as E3 ligases targeting ALDH1B1 (Figure 4E). As we predicted that ALDH1B1 is ubiquitinated by at least two E3 ligases and one of them is capable of targeting ALDH1B1 to proteasomal degradation in an AMBRA1-independent manner, we expected that one of DDB1 and TRAF6 would be able to affect the protein stability of ALDH1B1. However, neither of them showed an effect when transfected into HCT116 cells stably expressing Myc-tagged ALDH1B1 (Supplementary Figure S2). These results suggested that ALDH1B1 ubiquitination by DDB1 and TRAF6 may not be involved in proteasomal degradation. Based on the data, it is safe to say that proteasomal degradation of ubiquitinated ALDH1B1 may be induced by an E3 ligase other than DDB1 and TRAF6 or may be independent of the ubiquitination system.

### 2.5. ALDH1B1 Is Ubiquitinated via Cooperation of AMBRA1 with TRAF6 and through K27, K29, and K33 Linkages

**Figure 5:**
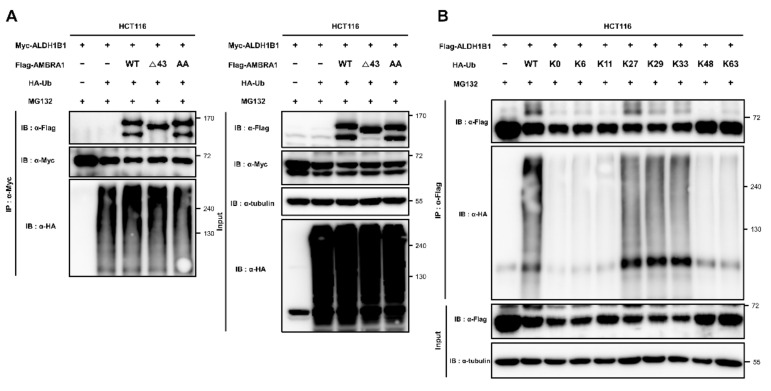
AMBRA1 ubiquitinates ALDH1B1 by mediating TRAF6, and ALDH1B1 is ubiquitinated by K27-, K29-, and K33-linked ubiquitin chains. (**A**) Ubiquitination of Myc-ALDH1B1 in AMBRA1 WT-, Δ43-, and AA-overexpressing cells as identified by Co-IP. Empty vector and Flag-AMBRA1 WT, Δ43, and AA were cotransfected with HA-Ub and Myc-ALDH1B1 into HCT116 cells. After treatment with 10 μM MG-132 for 6 h, immunoprecipitation and western blot analysis were performed (*n* = 3). (**B**) Using HA-Ub mutants (HA-Ub K0, K6, K11, K27, K29, K33, K48, and K63), the ubiquitination linkage types of Myc-ALDH1B1 were investigated by a cell-based ubiquitination assay (*n* = 3).

To identify the E3 ligase that is targeted to ALDH1B1 by AMBRA1, we constructed mutant forms of AMBRA1 that had lost the ability to bind to DDB1 or TRAF6. Two elegant studies have revealed the regions of AMBRA1 that are important for interaction with DDB1 or TRAF6. Amino acids 1–43 of AMBRA1 comprise the DDB1-binding region of AMBRA1 [18] and amino acids 618–623 and 681–686 are crucial for the AMBRA1–TRAF6 interaction [3]. Based on these data, we generated a deletion-mutant construct (AMBRA1 Δ43) and a double-mutant construct (AMBRA1 E620AE683A and AMBRA1 AA), and then their ability to ubiquitinate ALDH1B1 was evaluated in comparison with that of wild-type (WT) AMBRA1. As shown in Figure 5A, AMBRA1 AA could no longer ubiquitinate ALDH1B1, unlike AMBRA1 Δ43, suggesting that AMBRA1 mediates ALDH1B1 ubiquitination via interaction with TRAF6. To determine the linkage type of ALDH1B1 ubiquitination, mutant forms of pRK5-HA-Ub, in which 6 out of 7 lysine (K) residues of ubiquitin were mutated to arginine to establish a specific ubiquitination linkage type (HA-Ub K6, K11, K27, K29, K33, K48, or K63) or all 7 lysine residues were altered (HA-Ub K0), were used for in vivo ubiquitination assays. The results showed that the ubiquitination of ALDH1B1 by HA-Ub K27, K29, and K33 was comparable to that by WT HA-Ub, whereas the other mutants had lost their ubiquitination capacity (Figure 5B). These data were supported by previous studies reporting that TRAF6 is capable of promoting K6-, K27-, K29-, and K33-mediated ubiquitination, apart from canonical K63 assembly [27,28].

### 2.6. AMBRA1 Ubiquitinates ALDH1B1 through K27 and K33 Linkages by Cooperating with Different Partners

**Figure 6:**
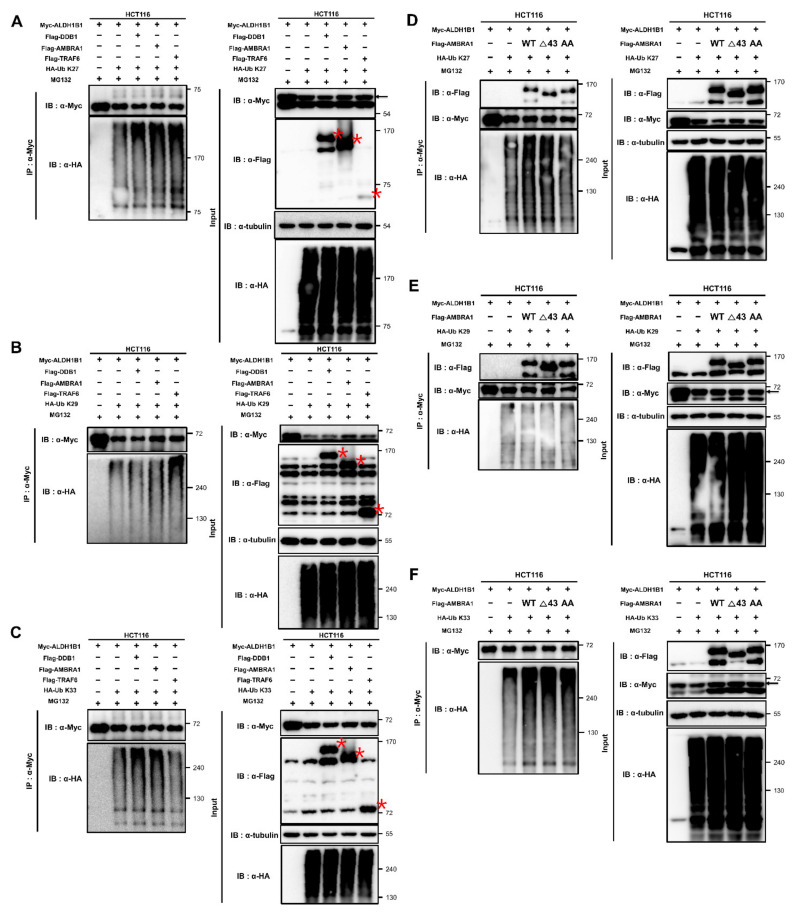
ALDH1B1 ubiquitination by AMBRA1 is mediated by K27 and K33 linkages and by different E3 ubiquitin ligases. (**A**–**C**) Anti-Myc immunoprecipitates were obtained from HCT116 cells transfected with Myc-ALDH1B1, three ALDH1B1-ubiquitinating factors (Flag-AMBRA1, Flag-DDB1, and Flag-TRAF6) and one of three ALDH1B1-ubiquitinating HA-Ub mutants (HA-Ub K27, K29, or K33). Cells were incubated with 10 µM MG132 for 6 h before cell lysis. Immunoprecipitates were immunoblotted with an anti-HA antibody to analyze the ubiquitination of ALDH1B1 and the specific linkage type (*n* = 3). The red asterisks indicate the overexpressed proteins. (**D**–**F**) HCT116 cells were cotransfected with Myc-ALDH1B1, three Flag-AMBRA1 constructs (WT, ×43, and AA) and one of three ALDH1B1-ubiquitinating HA-Ub mutants (HA-Ub K27, K29, or K33). Cells were treated with MG132 (10 µM) for 6 h before cell lysis. ALDH1B1 ubiquitination was detected by immunoprecipitation and immunoblot analysis (*n* = 3).

The above data showed that ALDH1B1 is ubiquitinated by three proteins, AMBRA1, DDB1, and TRAF6 (Figure 4E), and ALDH1B1 is the target of three noncanonical ubiquitination types, K27-, K29-, and K33-linked ubiquitination (Figure 5B). To analyze its ubiquitination in depth, we conducted an in vivo ubiquitination assay to identify the ALDH1B1 ubiquitination factor(s) responsible for each ubiquitination linkage type. AMBRA1, DDB1, and TRAF6 induced upregulation of the K27-mediated ubiquitination of ALDH1B1 (Figure 6A). While K29-linked ALDH1B1 ubiquitination was considerably induced only by TRAF6 (Figure 6B), K33-linked ubiquitination was induced by AMBRA1 and DDB1 (Figure 6C). Since AMBRA1-mediated ALDH1B1 ubiquitination using WT HA-Ub depends on the interaction with TRAF6 (Figure 5A), we investigated whether AMBRA1 required TRAF6 for the K27- or K33-linked ubiquitination of ALDH1B1, using the AMBRA1 mutants shown in Figure 5A. Consistent with the above findings (Figure 5B), WT and mutant AMBRA1 were not able to ubiquitinate ALDH1B1 using HA-Ub K63, although the typical ubiquitination linkage type of TRAF6 is K63 linkage (Supplementary Figure S3). Further, the results showed that interaction with TRAF6 was necessary for AMBRA1 to ubiquitinate ALDH1B1 via K27-linked ubiquitin chains (Figure 6D). We next elucidated that none of the AMBRA1 constructs was capable of affecting K29-linked ALDH1B1 ubiquitination (Figure 6E), and that AMBRA1 did not depend on DDB1 or TRAF6 for K33-mediated ALDH1B1 ubiquitination (Figure 6F). Collectively, these data suggested that AMBRA1 is involved in K27- and K33-linked ALDH1B1 ubiquitination in cooperation with different partners.

### 2.7. K506, K511, and K515 Are Potential K27- and K33-Mediated Ubiquitination Acceptor Sites of ALDH1B1 Involved in the Self-Association of ALDH1B1

**Figure 7:**
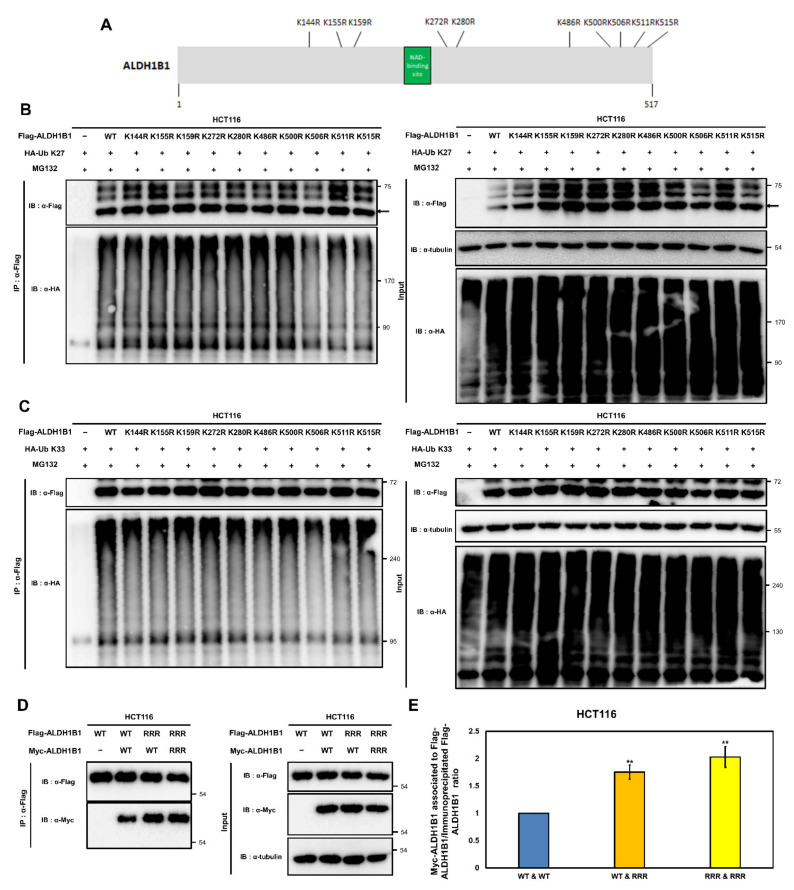
Identification of K27- and K33-mediated ubiquitination acceptor sites within ALDH1B1 and its effect on the self-association of ALDH1B1. (**A**) Schematic diagram showing point mutations in the predicted ubiquitination sites of ALDH1B1. (**B**,**C**) K27- and K33-linked ubiquitination of ALDH1B1 WT and single lysine-mutated forms (K144R, K155R, K159R, K272R, K280R, K486R, K500R, K506R, K511R, and K515R) were investigated by immunoprecipitation. Flag-tagged WT ALDH1B1 or ALDH1B1 mutants were cotransfected with HA-tagged ubiquitin K27 or K33 into HCT116 cells. Cells were treated with 10 µM MG132 for 6 h and protein lysates were analyzed by immunoprecipitation and western blot analysis (*n* = 3). (**D**) The self-association of WT and a triple mutant construct of ALDH1B1 was analyzed by Co-IP (*n* = 4). (**E**) Quantification of the results in (**D**) using ImageJ. ** *p* < 0.01.

The above data indicated that AMBRA1 suppresses the assembly of the ALDH1B1 enzymatic functional unit and that AMBRA1 ubiquitinates ALDH1B1 with K27- and K33-linked ubiquitin chains. Based on these data, we hypothesized that the suppressive effect of AMBRA1 on ALDH1B1 self-association is correlated with its ability to ubiquitinate ALDH1B1. To verify this hypothesis, we identified the lysine residues of ALDH1B1 that are ubiquitinated through K27- and K33-linked ubiquitin chains and examined whether they affect the self-association of ALDH1B1. According to a structural modeling study of ALDH1B1 [19], 10 lysine residues of ALDH1B1, i.e., K144, K155, K159, K272, K280, K486, K500, K506, K511, and K515, are involved in the formation of the ALDH1B1 tetramer. More specifically, K144, K159, and K515 make salt bridges across the ALDH1B1 tetramer subunits, K155, K159, and K486 are involved in the formation of hydrogen bonds across the ALDH1B1 tetramer interfaces, and K272, K280, K500, K506, and K511 are the interfacial residues presenting at the tetramer contact area. Based on this information, we generated mutant ALDH1B1 constructs where each lysine was changed to an arginine through single point mutagenesis (Figure 7A). Then, we examined whether these point mutations had an effect on K27- or K33-mediated ALDH1B1 ubiquitination as AMBRA1 was shown to ubiquitinate ALDH1B1 with K27- and K33-linked ubiquitin chains (Figure 6). In a ubiquitination assay using HA-Ub K27, the ubiquitination of the ALDH1B1 K506R, K511R, and K515R mutants was markedly reduced when compared with that of WT ALDH1B1 (Figure 7B). Next, the K33-linked ubiquitination of ALDH1B1 mutants was analyzed, and the results revealed that ALDH1B1 K506R was less ubiquitinated than the other ALDH1B1 mutant constructs (Figure 7C). These data suggested that K506, K511, and K515 are important for the K27-linked ubiquitination of ALDH1B1, whereas K33-linked ubiquitination may occur at K506.

Next, we evaluated the roles of the potential K27- and K33-mediated ubiquitination sites of ALDH1B1 in its self-association behavior. We generated triple point mutations at K506, K511, and K515 of ALDH1B1 and termed the construct Flag [Myc]-ALDH1B1-RRR. In in vivo ubiquitination assays, the ubiquitination of Flag [Myc]-ALDH1B1-RRR was not affected by the ectopic expression of AMBRA1 (Supplementary Figure S4). Then, we performed Co-IP using WT ALDH1B1 or ALDH1B1-RRR. The results showed that when ALDH1B1 had resistance to ubiquitination by AMBRA1, the self-association of ALDH1B1 was increased (Figure 7D,E). This suggested that AMBRA1 negatively regulates the self-association of ALDH1B1 by ubiquitinating the residues involved in ALDH1B1 tetramer formation.

## 3. Discussion

The current study provided novel insights into the tumor-suppressive mechanism of AMBRA1 and the post-translational modification of ALDH1B1, a CSC marker. AMBRA1 is a novel substrate receptor protein that interacts with and ubiquitinates ALDH1B1. The cooperation of AMBRA1 with E3 ligases such as TRAF6 is required for the K27- and K33-linked poly-ubiquitination of ALDH1B1. The AMBRA1-mediated ubiquitination of ALDH1B1 reduces the self-association of ALDH1B1 and the transcription of *PTEN*, which is associated with ALDH1B1 enzyme activity, and is followed by downregulation of the CSC-associated Wnt/β-catenin pathway. Collectively, our data demonstrate for the first time that ALDH1B1 is a novel target of AMBRA1, a substrate receptor protein of the ubiquitin conjugation system, and that the AMBRA1-mediated noncanonical ubiquitination of ALDH1B1 is involved in the regulation of ALDH1B1 self-association.

Accumulating evidence supports that not all tumor cells are equal and that CSCs, a subpopulation of tumor cells that have self-renewal capability, fuel tumor growth [29]. In colorectal cancer, CSCs have been associated with poor prognosis and low survival rates; furthermore, they contribute to increased relapse and tumor metastatic ability in colorectal cancer patients [30]. A recent study revealed that genes associated with intestinal stem cells could be used to predict relapse in colorectal cancer patients [31], one of which was *ALDH1B1*, a well-known marker of intestinal stem cells and colorectal CSCs [13]. In addition, the depletion of ALDH1B1 in colon cancer cells affected the Wnt/β-catenin pathway [15], which has central roles in normal stem cells and CSCs [24]. However, the post-translational modifications (PTMs) and regulation of ALDH1B1 remain largely unexplored. In the present study, we showed that ALDH1B1 enzyme activity is negatively regulated by AMBRA1-mediated K27- and K33-linked ubiquitination. Following recent reports that ubiquitination is highly prevalent in CSCs [26], our data point out that ALDH1B1 is a new target that is regulated by ubiquitination. Besides ubiquitination, other PTMs can modify protein functions. Therefore, further studies are necessary to find out whether ALDH1B1 is also regulated by other PTMs and the crosstalk between them.

In this study, several novel protein–protein interactions were identified by Co-IP assays. The interaction between AMBRA1 and ALDH1B1, which had only been suggested by proteomics data [18], was verified in this study by immunoprecipitation and immunoblot analyses. Although the self-association of ALDH1B1 had been suggested by computational modeling [19], we provided cell assay-based evidence of ALDH1B1 self-association on the basis of a previous study that showed self-association of ULK1 using differentially tagged ULK1 [3]. Furthermore, for the first time, two E3 ligases, DDB1 and TRAF6, were shown to form a complex with and ubiquitinate ALDH1B1.

Homotypic polyubiquitination is mediated by the N-terminal methionine (M1) or seven Lys residues (K6, K11, K27, K29, K33, K48, K63) of ubiquitin, and each polyubiquitination linkage type has a distinctive conformation and a unique role [32]. Among the homotypic polyubiquitin chains, K48- and K63-linked ubiquitin chains, which are referred to as canonic polyubiquitin chains, exert several functions, such as proteasomal degradation and signal transduction. In contrast, the other ubiquitination linkage types, which are noncanonical, require further investigation. We showed that ALDH1B1 is modified by the noncanonical K27-, K29-, and K33-linked chains. The K27- and K33-mediated ubiquitination of ALDH1B1 inhibits the self-interaction of ALDH1B1. The role of the K29-linked ubiquitination of ALDH1B1 remains to be elucidated.

In summary, we verified that AMBRA1 negatively regulates the self-association of ALDH1B1 via K27- and K33-linked ubiquitination. AMBRA1 downregulates the CSC-associated Wnt/β-catenin pathway via ALDH1B1. Moreover, our data revealed that ALDH1B1 is ubiquitinated by TRAF6 in an AMBRA1-independent way through K29-linked ubiquitin chains, and by DDB1 through K27- and K33-mediated ubiquitin chains.

Many questions are left after this study. The function of the K29-linked ubiquitination of ALDH1B1 remains to be elucidated. Whether the K29-mediated ubiquitination of ALDH1B1 by TRAF6 requires another substrate receptor, whether TRAF6 can directly ubiquitinate ALDH1B1 without a substrate receptor, and whether AMBRA1 determines the ubiquitination linkage type as well as the specific substrate of the E3 ubiquitin ligase remain unknown. Finally, the detailed mechanism of K27- and K33-linked ubiquitination to inhibit the self-association of ALDH1B1 remains to be unraveled.

## 4. Materials and Methods

### 4.1. Cell Lines and Culture

HCT116 human colorectal cancer cells and the human embryonic kidney cell lines HEK293T and HEK293FT were used. Among several colorectal cancer cell lines, HCT116 was selected for the following reasons. According to a previous study [23], ALDH1A1 activity is highly associated with the SC properties of some tumors. As with ALDH1B1, ALDH1A1 contributes to cancer stemness, metastasis, and chemoresistance by generating retinoic acids. To prevent any confounding effect of ALDH1A1, we decided to evaluate ALDH1B1 activity in HCT116 cells, which reportedly show high ALDH1B1 expression and very low ALDH1A1 expression [22].

HCT116 cells were cultured in RPMI-1640 medium (LM011-01; Welgene, Gyeongsan, Korea) supplemented with fetal bovine serum (EF-0500-A, Atlas Biologicals, Fort Collins, CO, USA) and 1× penicillin-streptomycin solution (30-002-Cl, Corning, Mediatech Inc., Manassas, VA, USA). HEK293T and HEK293FT cells were cultured in Dulbecco’s modified Eagle’s medium (LM001-05, Welgene, Gyeongsangbuk-do, Korea) with the same supplements. For ALDH1B1- and AMBRA1-knockdown HCT116 cells, 2 μg/mL puromycin (puromycin dihydrochloride; Santa Cruz Biotechnology, Dallas, TX, USA) was added to the medium as the selection agent. For HCT116 cells transfected with pCMV-Myc-ALDH1B1, 600 ng/mL G418 (G418 disulfate salt; Sigma-Aldrich, St. Louis, MO, USA) was added to the medium for the selection of stably ALDH1B1-overexpression cells. For all cell culturing, standard conditions (5% CO_2_ and 37 °C) were used.

### 4.2. Cloning and Site-Directed/Deletion Mutagenesis

We used the pCMV-3Tag-6 plasmid vector (Addgene, Teddington, UK), which is a mammalian expression vector, to tag proteins with the 3×Flag epitope at the N-terminus, and pCMV-3Tag-2 (Addgene, Teddington, UK) to tag proteins with the 3×Myc epitope at the N-terminus. cDNA, which was reverse transcribed from RNA extracted from HCT116 cells using oligo-dT primers (Thermo Fisher Scientific, Waltham, MA, USA), was used as the template to amplify the coding sequences of target genes. The primer sets used are listed in Supplementary Table S1. The amplified target gene sequences were cloned into plasmid vectors using appropriate restriction enzymes (Enzynomics, Daejeon, Korea) and a DNA ligation kit (Takara Bio, Shiga, Japan) according to the manufacturers’ protocols. For site-directed/deletion mutagenesis, plasmids containing wild-type target genes were used as a template to amplify mutant target genes. The primers used for mutagenesis are listed in Supplementary Table S2. Wild-type and mutant ubiquitins cloned into the pRK5 vector (pRK5-HA-Ub WT (Addgene plasmid 17608), K0(17603), K6(22900), K11(22901), K27(22902), K29(22903), K33(17607), K48(17605), K63(17606)) were kindly provided by Drs. Ted Dawson and Sandra Weller.

### 4.3. Transfection of Plasmid Constructs and Infection of Viral Particles

Opti-MEM (Thermo Fisher Scientific, Waltham, MA, USA) and Lipofectamine 2000 transfection reagent (Invitrogen, Grand Island, NY, USA) were used for transgene transfection. A mixture of plasmids and transfection reagent (1:2) was prepared in 1 mL of serum-free Opti-MEM. After incubation for the liposomes to entrap DNA plasmids, we carefully added the mixture into culture medium. For lentiviral infection, cells were cultured in medium containing the lentiviral particles and polybrene at a final concentration of 8 μg/mL (hexadimethrine bromide, Sigma-Aldrich, St. Louis, MO, USA). After 6 h, the culture medium was replaced with fresh medium. For cell selection, appropriate drugs were added 24 h later.

### 4.4. Construction of AMBRA1- and ALDH1B1-Knockdown Cell Lines

AMBRA1- and ALDH1B1-depleted HCT116 cells were generated using shRNA-based gene silencing. First, shRNAs were cloned into a pLKO.1 TRC cloning vector (Addgene, Teddington, UK). The shRNAs used, targeting the coding sequences and 3′-untranslated regions, are listed in Supplementary Table S3. shRNAs targeting firefly luciferase genes were used as a control. To produce lentiviral particles, 2.25 μg pMD2.G envelope plasmid, 6.75 μg psPAX2 packaging plasmid, and 7 μg shRNAs in pLKO.1 puro vector were cotransfected into HEK293FT cells. After harvesting of the lentiviral particles, they were used for infection as described above.

### 4.5. Generation of ALDH1B1 Stable Overexpression Cell Lines

To construct a HCT116 cell line stably overexpressing ALDH1B1, we inserted *ALDH1B1* into pCMV-3Tag-2 and transfected the plasmid into HCT116 cells. After 48 h, the cells were selected on G418 (600 ng/mL). At least 10 days later, the expression efficiency was evaluated by RT-qPCR and western blotting with anti-Myc antibody.

### 4.6. Immunoblot Analysis

Harvested cells were resuspended in radioimmunoprecipitation assay buffer (1% Nonidet P-40 (NP-40), 150 mM NaCl, 50 mM Tris-HCl, pH 8.0, 0.5% sodium deoxycholate, 0.1% sodium dodecyl sulfate (SDS)) supplemented with protease inhibitors (1 mM NaF, 2 mM Na_3_VO_4_, 1 mM PMSF, and 1× PI (11873580001, Roche, Basel, Switzerland)). Proteins were collected by centrifugation (13,200 rpm, 4°C, 25 min) and quantified using Protein Assay Dye Reagent Concentrate (Bio-Rad, Hercules, CA, USA) and a Sunrise Microplate Reader (Sunrise-Basic Tecan, Tecan Austria GmbH, Grödig, Austria). The total proteins were separated by molecular mass using electrophoresis and transferred to polyvinylidene fluoride membranes (EMD Millipore, Billerica, MA, USA). The membranes were blocked using skim milk (BD, Franklin Lakes, NJ, USA) or bovine serum albumin (GenDEPOT, Katy, TX, USA) and then incubated with primary antibodies under agitation at 4 °C overnight and then secondary antibodies at room temperature for 1–2 h. For detection, we used an ImageQuant^TM^ LAS 4000 instrument (GE Healthcare, Bronx, NY, USA), and ImageJ (1.53k14, Bethesda, MD, USA) was used for quantification.

### 4.7. Co-Immunoprecipitation Assay

Cells were lysed in 1% NP-40 buffer (1% NP-40, 10% glycerol, 50 mM Tris-HCl, pH 7.4, 5 M NaCl) supplemented with protease inhibitors. Proteins were collected by centrifugation and incubated with anti-c-Myc agarose affinity gel antibody (Sigma-Aldrich, St. Louis, MO, USA) or anti-Flag affinity gel (Sigma-Aldrich, St. Louis, MO, USA) at 4 °C overnight under rotation. Then, the beads were washed with lysis buffer five times. Protein complexes were eluted by boiling with Laemmli SDS sample buffer (GenDEPOT, Texas, USA) and analyzed by immunoblot analysis.

### 4.8. Cell-Based Ubiquitination Assay and MG132 Treatment

The ubiquitination of ALDH1B1 in the cells was investigated using a cell-based ubiquitination assay. After treating the cells with 10 μM MG132 (proteasome inhibitor; Sigma-Aldrich, St. Louis, MO, USA) for 6 h, they were harvested and lysed using a ubiquitination assay buffer (1% NP-40, 150 mM NaCl, 1 mM EDTA, 2.5 mM MgCL_2_, 50 mM Hepes, pH 7.4, and 0.5% sodium deoxycholate) supplemented with 0.1% SDS, 10 mM N-ethylmaleimide (deubiquitinase inhibitor; Sigma-Aldrich, St. Louis, MO, USA), and protease inhibitors. The proteins were collected and immunoprecipitated using an anti-c-Myc agarose affinity gel antibody and immunoblotted using an anti-HA antibody.

### 4.9. RT-qPCR

Total RNA was extracted using TRI reagent (TR118-200; MRC, Cambridge, UK) and chloroform (Merck, Kenilworth, NJ, USA) and reverse transcribed into cDNA using a RevertAid First Strand cDNA Synthesis Kit (K1622; Thermo Fisher Scientific, Waltham, MA, USA) with oligo-dT primers. qPCRs were run using SYBR Premix EX^TM^ Taq II (RR82LRB; Takara Bio, Shiga, Japan) on a 7300 Real-time PCR system (Applied Biosystems, Franklin Lakes, NJ, USA). Dissociation curves were generated to test the presence of off-target amplification products or contaminants. The primers used are listed in Supplementary Table S4.

### 4.10. Statistical Analysis

Data are reported as means ± standard deviations (SDs). For pairwise comparisons, we used the two-tailed Student’s *t*-test. *p* < 0.05 was considered significant.

## 5. Conclusions

This study revealed that AMBRA1 acts as a negative regulator of ALDH1B1 by controlling its ubiquitination. The K27- and K33-linked poly-ubiquitination of ALDH1B1 is catalyzed by AMBRA1 in cooperation with E3 ligases, such as TRAF6. The AMBRA1-mediated ubiquitination of ALDH1B1 results in reduced self-association of ALDH1B1, leading to downregulation of the CSC-associated Wnt/β-catenin pathway. This study provided the first evidence of the important role of AMBRA1-mediated noncanonical ubiquitination in the negative regulation of the CSC marker ALDH1B1. Thus, this study may contribute to the development of CSC-selective therapeutics by identifying the AMBRA1-ALDH1B1-Wnt/β-catenin axis as a novel target of CSC function.

## Data Availability

Not applicable.

## Supplementary Materials

The following are available online at https://www.mdpi.com/article/10.3390/ijms222112079/s1, Figure S1: Efficiencies of AMBRA1 overexpression and *ALDH1B1* knockdown were confirmed by RT-qPCR (corresponds to Figure 3) (*n* = 3); Figure S2: Protein level of Myc-ALDH1B1 after overexpressing Flag-DDB1 and Flag-TRAF6; Figure S3: K63-mediated ubiquitination of Myc-ALDH1B1 as investigated by co-immunoprecipitation; Figure S4: The ubiquitination of Myc-ALDH1B1-RRR (Myc-ALDH1B1 K506R, K511R, K515R) was analyzed after ectopic expression of AMBRA1; Table S1: Primer sequences used for cloning target genes; Table S2: Primer sequences used for mutagenesis; Table S3: Primer sequences used for small hairpin RNA(shRNA)-based knockdown system; Table S4: Primer sequences used for real-time PCR.

## Abbreviations

CSC, cancer stem cell; HA-Ub, HA-tagged ubiquitin; IP, immunoprecipitated; KD, knockdown.
