# Supplementary material for: AMBRA1 Negatively Regulates the Function of ALDH1B1, a Cancer Stem Cell Marker, by Controlling Its Ubiquitination
Source: Int J Mol Sci. 2021 Nov 8;22(21):12079. doi: 10.3390/ijms222112079 (PMC8584921; doi:10.3390/ijms222112079)
Supplement: Supplementary file 1 [file ijms-22-12079-s001.zip › ijms-1440258-supplementary.pdf]

AMBRA1 negatively regulates the function of ALDH1B1, a Cancer Stem Cell Marker, by Controlling its Ubiquitination

Seung-Heon Baek [1,2], Yeun-Kyu Jang [1,2,*]

[1] Department of Systems Biology, College of Life Science and Biotechnology, Yonsei University, Seoul 03722, Republic of Korea; baeksh9505@naver.com
[2] BK21 Yonsei Education & Research Center for Biosystems, Yonsei University, Seoul 03722, Republic of Korea.
* Correspondence: ykjang@yonsei.ac.kr, Tel: 82-2-2123-5654, Fax: 82-2-312-5657

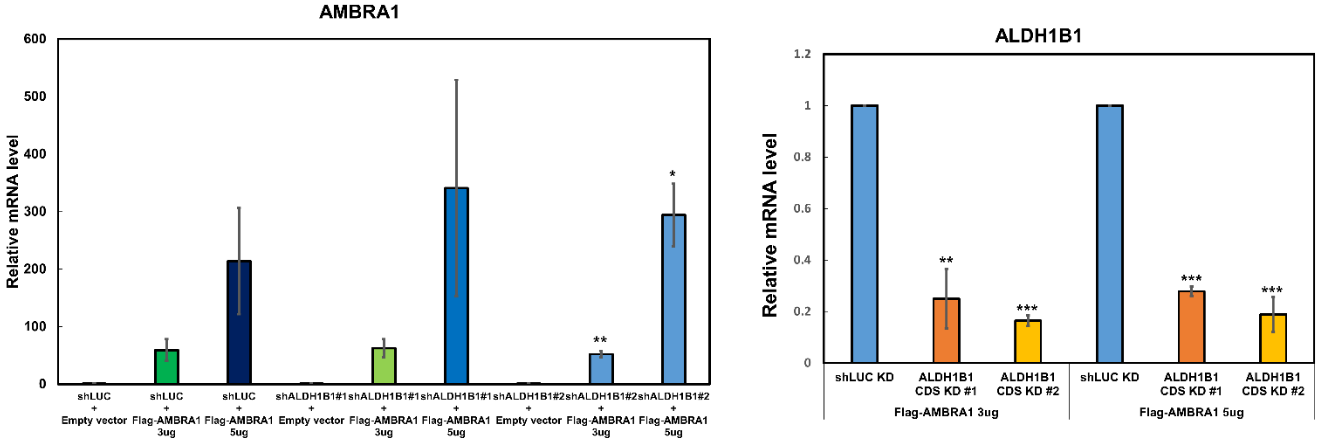

Supplementary Figure S1. Efficiencies of AMBRA1 overexpression and *ALDH1B1* knockdown were confirmed by RT-qPCR (corresponds to Figure 3) (n = 3).

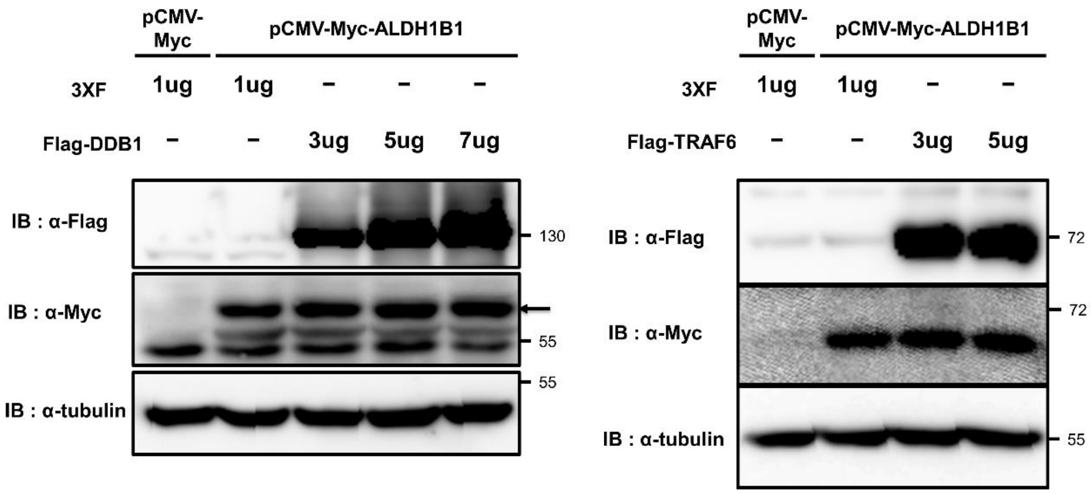

Supplementary Figure S2. Protein level of Myc-ALDH1B1 after overexpressing Flag-DDB1 and Flag-TRAF6. Indicated amounts of Flag-DDB1 or Flag-TRAF6 were transfected into pCMV-Myc-ALDH1B1 HCT116 cells and the protein level of Myc-ALDH1B1 was investigated by western blot analysis. α-Tubulin was detected as a loading control (n = 3).

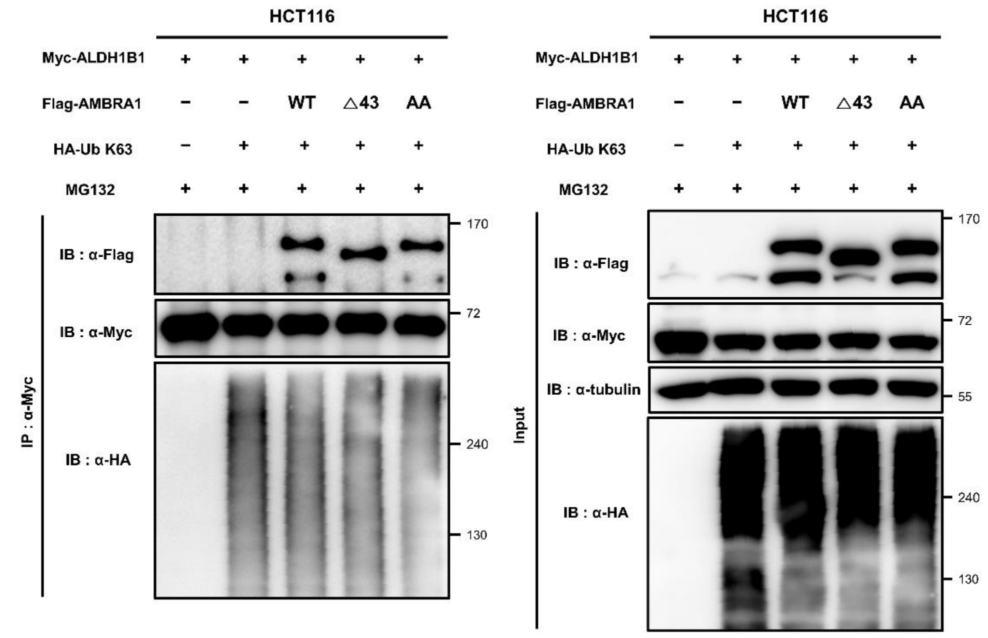

Supplementary Figure S3. K63-mediated ubiquitination of Myc-ALDH1B1 as investigated by co-immunoprecipitation. Myc-ALDH1B1 and Flag-AMBRA1 WT, △43, AA were cotransfected with HA-Ub K63 into HCT116 cells, which were treated with 10 μM MG-132 for 6 h. K63-linked ubiquitination of Myc-ALDH1B1 was detected by immunoprecipitation with anti-c-Myc agarose affinity gel antibody (*n* = 3).

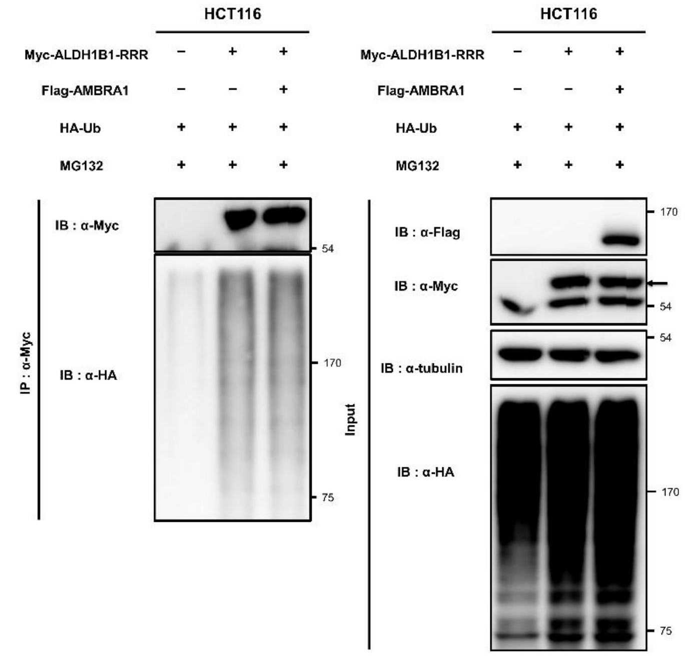

Supplementary Figure S4. The ubiquitination of Myc-ALDH1B1-RRR (Myc-ALDH1B1 K506R, K511R, K515R) was analyzed after ectopic expression of AMBRA1. As shown in Fig. 4A, we observed an increase in the ubiquitination of wild-type Myc-ALDH1B1 by AMBRA1. In contrast, immunoprecipitation and immunoblot analysis confirmed that

potential K27- and K33-linked ubiquitination-defective ALDH1B1 interacted with a similar amount of HA-Ub before and after AMBRA1overexpression (n = 3).

Supplementary Table S1. Primer sequences used for cloning

Gene	Primer sequence (5′ to 3′)	Accession no.
**AMBRA1**	F : GCGGATATCATGAAGGTTGTCCCAGAA<br>R : GCGATCGATCTACCTGTTCCGTGGTTC	NM_001367471.1
**ALDH1B1**	F : ATAGATATCATGCTGCGCTTCCTGGCA<br>R : GCGGTCGACTTACGAGTTCTTCTGAGG	NM_000692.5
**DDB1**	F : ATAGATATCATGTCGTACAACTACGTGGTA<br>R : ATAGTCGACCTAATGGATCCGAGTTAGCTC	NM_001923.5
**TRAF6**	F : GCGAAGCTTATGAGTCTGCTAAACTGT<br>R : ATAGTCGACCTATACCCCTGCATCAGT	NM_145803.3

Supplementary Table S2. Primer sequences used for mutagenesis

Mutation	Primer sequence (5′ to 3′)	Type of mutation
AMBRA1 △43	F : GCGGATATCATGGGCAAGAGAGTAGAACTG<br>R : GCGATCGATCTACCTGTTCCGTGGTTC	Deletion
AMBRA1 E620A	F : AGTTGCCACCTCTCGCGCGGACTGAGGGCCA<br>R : TGGCCCTCAGTCCGCGCGAGAGGTGGCAACT	Point mutation
AMBRA1 E683A	F : AGGATATGCCTGAGGCGAGCTCTGAGGAGGA<br>R : TCCTCCTCAGAGC31merTCGCCTCAGGCATATCCT	Point mutation

Supplementary Table S3. Primer sequences used for small hairpin RNA(shRNA)-based knockdown system

Gene	Primer sequence (5′ to 3′)	Target region	Accession no.
**AMBRA1**	F : CCGG GTCCACGCTCTACCTTCTTAT CTCGAG ATAAGAAGGTAGAGCGTGGAC TTTTTG<br>R : AATTCAAAAA GTCCACGCTCTACCTTCTTAT CTCGAG ATAAGAAGGTAGAGCGTGGAC	**CDS (coding sequence)**	**NM_001367471.1**
	F : CCGG GCATGTGGACTCTTAACTGTA CTCGAG TACAGTTAAGAGTCCACATGC TTTTTG<br>R: AATTCAAAAA GCATGTGGACTCTTAACTGTA CTCGAG TACAGTTAAGAGTCCACATGC	**3′-UTR (3′ Untranslated Region)**	
**ALDH1B1**	F : CCGG GAATCCATCTACAATGAGTTT CTCGAG AAACTCATTGTAGATGGATTC TTTTTG<br>R : AATTCAAAAA GAATCCATCTACAATGAGTTT CTCGAG AAACTCATTGTAGATGGATTC	**CDS**	**NM_000692.5**
	F : CCGG TCGAGAGAACCGTGGAGAAAG CTCGAG CTTTCTCCACGGTTCTCTCGA TTTTTG<br>R : AATTCAAAAA TCGAGAGAACCGTGGAGAAAG CTCGAG CTTTCTCCACGGTTCTCTCGA	**CDS**	

Supplementary Table S4. Primer sequences used for real-time PCR

Gene	Primer sequence (5′ to 3′)	Accession no.
GAPDH	F : TGATGACATCAAGAAGGTGGTGAAG<br>R : TCCTTGGAGGCCATGTGGGCCAT	NM_001289746.2
β-actin	F : GCCCTGAGGCACTCTTCCA<br>R : CCAGGGCAGTGATCTCCTTCT	NM_001101
AMBRA1	F : GAAATGGGAGGGCAAGAGA<br>R : TCACATGGGTGGAGGCTAA	NM_001367471.1
ALDH1B1	F : AGAGGGCCAACAACACCA<br>R : CCCTCCCGTTTCCAGATT	NM_000692.5
PTEN	F : TGTTCAGTGGCGGAACTTG<br>R : TCGTGTGGGTCCTGAATTG	NM_001304718.2
CTNNB1	F : AGTTGATGGGCTGCCAGAT<br>R : TCAAACCAGGCCAGCTGAT	NM_001904.4
LGR5	F : CCTGTCCTTGCCTGTGCT<br>R : CCACCCTGAGCAACATCC	NM_003667.4
HIF1a	F : GGCAGCAACGACACAGAA<br>R : TTGAGTGCAGGGTCAGCA	NM_001530.4
ALDH1A3	F : TCTCGACAAAGCCCTGAAGT<br>R : GGCCAAAGCGTATTCACCTA	NM_000693.4
CD24	F : CCCACGCAGATTTATTCCAG<br>R : ACCACGAAGAGACTGGCTGT	NM_013230.3
SOX4	F : AAGTACCGGCCCAGGAAG<br>R : CTGCCACCGACCTTGTCT	NM_003107
